# Biodegradation of Poly(butylene succinate) Powder in a Controlled Compost at 58 °C Evaluated by Naturally-Occurring Carbon 14 Amounts in Evolved CO_2_ Based on the ISO 14855-2 Method

**DOI:** 10.3390/ijms10104267

**Published:** 2009-11-20

**Authors:** Masao Kunioka, Fumi Ninomiya, Masahiro Funabashi

**Affiliations:** National Institute of Advanced Industrial Science and Technology (AIST), Higashi 1-1-1, Tsukuba, Ibaraki 305-8565, Japan; E-Mails: f.ninomiya@aist.go.jp (F.N.); m.funabashi@aist.go.jp (M.F.)

**Keywords:** biodegradation, poly(butylenes succinate), ISO 14855-2, accelerator mass spectrometry, radiocarbon

## Abstract

The biodegradabilities of poly(butylene succinate) (PBS) powders in a controlled compost at 58 °C have been studied using a Microbial Oxidative Degradation Analyzer (MODA) based on the ISO 14855-2 method, entitled “Determination of the ultimate aerobic biodegradability of plastic materials under controlled composting conditions—Method by analysis of evolved carbon dioxide—Part 2: Gravimetric measurement of carbon dioxide evolved in a laboratory-scale test”. The evolved CO_2_ was trapped by an additional aqueous Ba(OH)_2_ solution. The trapped BaCO_3_ was transformed into graphite via a serial vaporization and reduction reaction using a gas-tight tube and vacuum manifold system. This graphite was analyzed by accelerated mass spectrometry (AMS) to determine the percent modern carbon [pMC (sample)] based on the ^14^C radiocarbon concentration. By using the theory that pMC (sample) was the sum of the pMC (compost) (109.87%) and pMC (PBS) (0%) as the respective ratio in the determined period, the CO_2_ (respiration) was calculated from only one reaction vessel. It was found that the biodegradabilities determined by the CO_2_ amount from PBS in the sample vessel were about 30% lower than those based on the ISO method. These differences between the ISO and AMS methods are caused by the fact that part of the carbons from PBS are changed into metabolites by the microorganisms in the compost, and not changed into CO_2_.

## 1. Introduction

**Figure 1.**
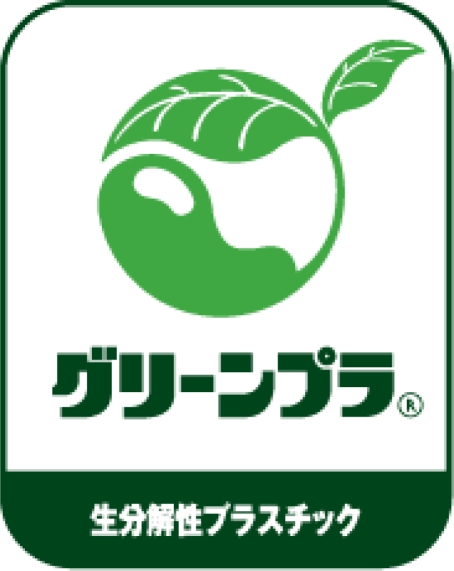
“Green Pla” mark for products made from biodegradable plastics authorized by the Japan BioPlastic Association (JBPA).

Biodegradable plastics are expected to be used for food containers which can be treated with food waste for composting and methane fermentation [1]. Food waste should be recycled to recover the carbon resources, such as compost or methane, as stated in a food recycling law by the Japanese legislation. If food packaging materials, such as a lunch box or container, are biodegradable, these materials’ waste is expected be changed to compost or methane in food waste treatment facilities. In addition, the materials used in the agricultural industries, such as mulching film or supported plastic products (support bars or nets for fruit or vegetable, etc.) can be waste-treated for composting mixed with agricultural waste. There is the mark certification system called “Green Pla” as shown in Figure 1, organized by Japan BioPlastic Association (JBPA) [2]. JBPA made a positive list related to biodegradable plastic products with use permission of the “Green Pla” mark. A company producing biodegradable plastic products can get JBPA’s permission, if the conditions for biodegradability and toxicity based on the International Standards (ISO) are at an acceptable level.

**Figure 2.**
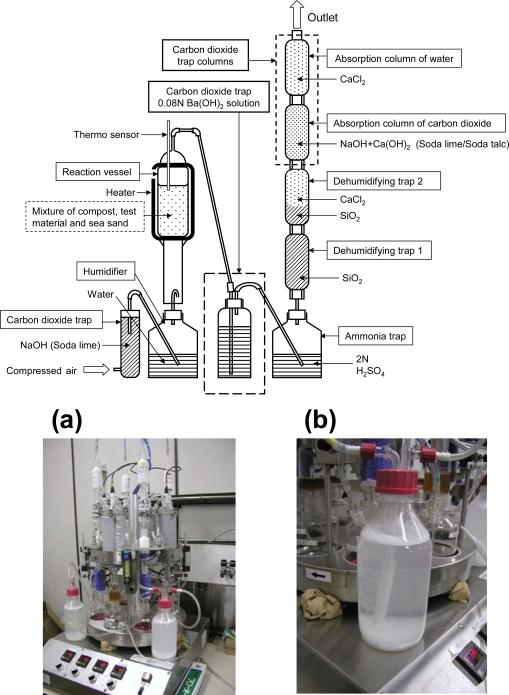
Biodegradation evaluation method by gravimetric measurement of carbon dioxide evolved in laboratory-scale test using Microbial Oxidative Degradation Analyzer (MODA) apparatus (a) in controlled compost at 58 °C based on ISO 14855-2. Additional carbon dioxide trap (Ba(OH)_2_ aqueous solution bottle (b)) was used only for the percent modern carbon (pMC) measurements by accelerated mass spectrometry (AMS) of evolved CO_2_ from poly(butylene succinate) (PBS) biodegradation.

**Figure 3.**
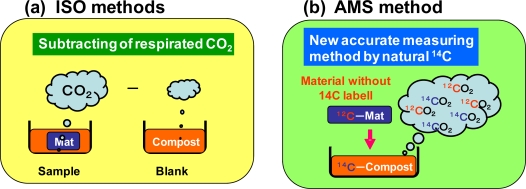
Evaluation of biodegradability based on evolved CO_2_ from bioplastic materials in compost.

The degrees of biodegradation can be determined by many methods. The simplest evaluation method of biodegradation is determining the remaining weight of the biodegraded samples removed from the test environment after a specific period. However, this method cannot accurately determine a degree of biodegradation over 70%, because the remaining samples of materials that have already biodegraded to 70% are small pieces and cannot be totally recovered. Their degrees of biodegradation should be higher than the true values. The degrees of biodegradation that range from 70 to 100% are very important for the quality control of products. Based on the ISO 14855-2 method [3], the CO_2_ evolved by the biodegradation process of the sample is collected in a simulated test environment (composting condition) using a gas sealed closed apparatus using reaction vessels with a CO_2_-free air supplier and CO_2_ trap system such as NaOH columns, as shown in Figure 2. The general ISO 14855-2 system has no Ba(OH)_2_ trap bottle [Figure 2 (b)] which is optionally used for BaCO_3_ collection in this study. This ISO method was used in this study to determine the degree of biodegradation in a controlled compost at 58 °C. The degree of biodegradation is a percentage of the evolved CO_2_ to the theoretical evolved CO_2_. In the case that all the sample, such as 10 g of poly(butylene succinate) (PBS) used in this study is biodegraded to CO_2_, the theoretical evolved CO_2_ is 20.47 g (10 g × 8CO_2_ (44 × 8)/PBS(172)). The evolved CO_2_ amount itself from a sample vessel cannot be used to calculate directly the degree of biodegradation, because this evolved CO_2_ value includes the respirated CO_2_ from the active and alive inoculums, such as the compost in the test environment as shown in Figure 3 (a). A blank vessel which includes only compost without the sample is necessary for the evaluation. From this blank vessel, the respirated CO_2_ value from the compost was obtained. Therefore, the degree of biodegradation is calculated as indicated in Equation (1):

(1) $$\text{Degree}\;\text{of}\;\text{biodegradation}=\frac{\text{total}\;\text{evolved}\;{\text{CO}}_{2}\;(\text{sample}\;\text{vessel})-\text{respired}\;{\text{CO}}_{2}\;(\text{blank}\;\text{vessel})}{\text{Theoretical}\;{\text{CO}}_{2}\;\text{value}\;\text{from}\;\text{sample}\;\text{material}}\times 100\;(\%)$$

According to ISO 14855-2, duplicate vessels are required for both sample and blank. Based on the ISO method, the respiration activities of the sample and blank vessels are regulated to be the same. However, these respiration values of the sample and blank are slightly different due to the increased respiration activity in the sample vessel after sufficient biodegradation of the sample. The total evolved CO_2_ amount increased with this increasing respiration. Therefore, the degree of biodegradation for the well biodegraded sample can rarely be over 100%.

There are methods which can separately measure the amounts of biodegradation and respiration from one vessel. These methods use the sample labeled by radiocarbon-14 (^14^C). By comparison of the ^14^C enriched ratio between the original sample and evolved CO_2_ measured by a liquid scintillation counter, the exact evolved CO_2_ from the biodegradation of the sample can be culculated. The evaluation method using ^14^C labeled materials has been studied by many researchers. Albertsson *et al*. studied the ^14^C labeled polyethylene degradation under simulated soil conditions for long time. (ca. 10 years) [4]. The American Society of Testing and Materials (ASTM) adopted the biodegradation evaluation methods using ^14^C labeled materials, which are ASTM D6340 [5], D6692 [6] and D6776 [7]. The anaerobic biodegradation of bioplastics, such as bacterial and synthetic polyesters, were studied by detecting ^14^CH_4_ and ^14^CO_2_ from radiolabeled compounds [8]. Recently, the methods using ^3^H (tritium) labeled compounds were developed [9,10]. These methods can obtain an accurate degree of biodegradation for labeled samples. However, for the general biodegradable product, it is very difficult to prepare the ^14^C of ^3^H-enriched sample with the same total sample composition and molecular weight as the product.

In our laboratory, we have developed a biodegradation evaluation method using naturally occurring ^14^ C in biomass carbon, as shown in Figure 3 (b) [11]. Biomass carbon includes a very small amount of ^14^C atoms with the ratio of 1 × 10^−12^ to ^12^C. On the other hand, chemicals, such as polymers from petroleum, include no ^14^C. By measuring the ^14^C amount in CO_2_ evolved from a sample vessel with petroleum-based polycaprolactone (PCL) in the compost, in which the component is biomass carbon, the evolved CO_2_ biodegraded from PCL in the compost can be calculated from the dilution ratio of the ^14^C and the total evolved CO_2_ amount from only the sample vessel. The very low ^14^C concentration of the biomass carbon can be measured by accelerator mass spectrometry (AMS). It is very difficult to measure the ^14^C concentration of natural biomass carbon using a liquid scintillation counter. AMS can measure the isotope carbon ratio of ^12^C, ^13^C and ^14^C by detecting these carbon isotope weights of the atoms. AMS can count the ^14^C carbon atom numbers. The AMS method has been developed as the carbon dating method to determine the age of historical or geological samples. Recently, this AMS is used for determining the biobased content in chemicals derived from biomass resources based on ASTM D6866 [12]. AMS measurements are used for determining the biomass carbon ratio in biomass products [13–17]. It was found that the AMS method for the evaluation of biodegradation can separately determine the evolved CO_2_ from respiration and biodegradation of PCL by calculating the pMC in the evolved CO_2_ from one sample vessel.

The poly(butylene succinate) (PBS) used in this study is a biodegradable aliphatic polyester developed by Japanese companies such as Showa Highpolymer Co., Ltd [18]. This company produced PBS with the brand name “Bionole”. PBS has flexible properties like polyethylene with a 120 °C melting point, and is suitable for use as a mulching film in agricultural fields [1]. At this moment, PBS is petroleum based. PBS will be a biobased polyester produced from biomass materials or fermentation products in the near future, as reported by Showa Highpolymer [19] and Mitsubishi Chemicals [20,21].

In this study, the degree of biodegradation of PBS powders (Av. 157.8 μm) has been studied in a controlled compost at 58 °C using the Microbial Oxidative Degradation Analyzer (MODA) with sample and blank vessels based on ISO 14855-2. In addition, the degree of biodegradation for PBS powders have been evaluated in a compost at 58 °C using MODA by measuring the ^14^C concentration in the evolved CO_2_ only from the sample vessel. The ^14^C concentrations in the evolved CO_2_ were measured by AMS to evaluate the respiration and biodegradation of CO_2_ evolved from the sample vessel.

## 2. Experimental Section

### 2.1. Materials

Poly(1,4-butylene succinate), extended with 1,6 diisocyanatohexane (PBS, Aldrich Chemical Co., Japan) was used as received. Cellulose powder of thin-layer chromatography grade with a particle size of less than 20 μm (cellulose microcrystalline; Merck, Germany), soda talc (sodium hydroxide on support, granulated to about 1.6–3 mm; Merck, Germany), soda lime (sodium hydroxide on support, small granules of about 1.5–3 mm; Kanto Chemical, Japan) and sea sand (sand washed, 425–850 mm; Wako Pure Chemical, Japan) were used as received.

The PBS powder was prepared from polymer pellets (ca. 5 mm) [22,23]. The polymer pellets were crushed using a rotating mechanical mixer with titanium blades (10,000 rpm, 3 L) with cooling by dry ice. Crushing was done 15 times for 3 min each with a 5-min interval to prevent overheating of the motor in the mixer. After drying under reduced pressure at room temperature, the PBS powder was separated using sieves of 60 mesh (250 μm) and 120 mesh (125 μm). Standard sieves with a guarantee were used. These sieves with the crude polymer powders were placed on a sieve vibrator and vibrated for 15 min. The average size of the obtained PBS powders was 157.8 °m determined by the average of at least 100 particles in photographs by optical microscopy. This PBS is petroleum-based polymer. Therefore, the pMC of this PBS powders was 0% by AMS.

The controlled compost (YK-6, Hissan Trading Co., Ltd., Japan) for the Microbial Oxidative Degradation Analyzer (MODA) based on ISO 14855-2 was prepared as follows. The waste material of used wood block for growing mushrooms and chicken droppings was composted for seven months. During this period, a mature compost was prepared. After preparation, this obtained compost was sieved using a 4.7 mesh (4 mm), kept at room temperature and prevented from drying. The properties of this compost are shown in Table 1. This compost can presently be kept at room temperature for a long time, at least four years.

Before using this compost, an activation step (preincubation) was required to recover the biological activities for the respiration and biodegradation by the microorganisms. The controlled compost was prepared by mixing 144 g of compost (60 g total dry solids) and 320 g of sea sand and its water content was controlled to over 80 wt% (the weight of the volatile solids of this compost without sea sand). This amount was for one reaction vessel. Preincubation was done once for the total amount of blanks and samples using a large container (5 L). Sea sand was added to create good homogeneous conditions and a better aerobic environment inside the compost. This compost for the activation step was mixed once a day, and the water content was adjusted to 65 wt% for 7 days at 58 °C.

### 2.2. Biodegradation Test by MODA Apparatus Based on ISO 14855-2 (ISO Method)

A biodegradation test was performed using the MODA apparatus (Hissan Trade Co., Ltd., Japan) in a controlled compost at 58 °C as shown in Figure 2. Two sample reactors were used in the same MODA apparatus test, one without Ba(OH)_2_ trap bottle (to carry out a traditional ISO 14855-2 test), while the second sample reactor was equipped with the trap system for the percent modern carbon (pMC) determination by accelerator mass spectrometry (AMS) method as detailed in Section 2.3. The polymer sample powder (10 g) was well mixed in the activated compost (144 g) with sea sand (320 g) and transferred to each sample reaction vessel. Compost with no sample was used as a blank to determine the respiration activity of this compost during the test period under the same conditions as in the sample vessel. The biodegradation tests were performed at 58 °C and a 10 mL/min air (CO_2_-free) flow rate for 74 days. The activated compost used in this study produced 84.6 and 89.5 mg CO_2_ per gram of volatile solids over the first 10 days as determined for two blank vessels, respectively. In almost all cases, the number of experimental replicates of the blank or sample was two (duplicate). For the AMS measurement, only one sample vessel was used. The produced CO_2_ amounts were measured once a day by measuring the weights of the absorption column for carbon dioxide and the absorption column for water. The percent of biodegradation was calculated from the produced CO_2_ amount using the subtracted respiration CO_2_ amount determined from a blank and the theoretically produced CO_2_ amount of the added sample. For example, 10 g of PBS produced 20.47 g of CO_2_ which was the theoretical amount for the 100% biodegradation. As recommended by ISO 14855-2, once a week, the sample and compost were well mixed, and the water content was controlled. When the absorbed CO_2_ amounts for the absorption columns reached 40% of the theoretical absorption capacity, and the chemicals (soda lime and soda talc) inside the columns were changed.

### 2.3. Biodegradation Test by MODA Apparatus with Carbon Dioxide Trap Including the Ba(OH)_2_ Aqueous Solution

To determine the percent modern carbon (pMC) of evolved CO_2_ produced during the biodegradation of PBS by accelerator mass spectrometry (AMS), the produced CO_2_ was trapped as BaCO_3_ in a Ba(OH)_2_ aqueous solution (0.08 N, 500 mL in a 1 L gas-tight glass bottle with a bubbling tube) between the reaction vessel and the ammonia trap in the MODA apparatus as indicated in Figure 2 based on ISO 14855-2. The produced CO_2_ from the reaction vessel including the sample and compost reacts with Ba(OH)_2_ and is converted to BaCO_3_. BaCO_3_ is insoluble in the Ba(OH)_2_ aqueous solution and precipitates. After the determined test period (3–14 days) for pMC (sample), the Ba(OH)_2_ aqueous solution is changed to a fresh one. To determine the pMC (compost) of the produced CO_2_ from the blank reaction vessel including no sample and the compost, BaCO_3_ was collected using the Ba(OH)_2_ trap bottle in the MODA apparatus during the 0–3-day test period. The precipitated BaCO_3_ was well mixed by gently shaking the trap several times and collected by filtration under reduced pressure. The solution bottles were tightly closed before filtration, and the filtration step was quickly carried out to avoid any additional reaction with CO_2_ in the room atmosphere. The collected BaCO_3_ was freeze-dried under reduced pressure at room temperature and weighed. This dried BaCO_3_ was used for the determination of pMC by AMS.

### 2.4. Graphite Preparation from BaCO_3_ for AMS Measurements

**Figure 4.**
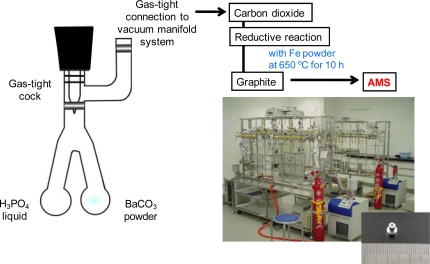
Gas-tight glass tube for sample preparation for AMS from trapped BaCO_3_ to CO_2_ by PBS biodegradation and pretreatment method to produce graphite from purified CO_2_.

The sample preparation and measurements were done at the Institute of Accelerator Analysis, Ltd. (IAA), Japan. All carbon atoms of the collected BaCO_3_ samples were transformed into graphite carbons through serial vaporization and reduction reactions using a gas-tight glass tube with a closing stopcock which can be connected to a vacuum manifold system as indicated in Figure 4 for the pMC measurement of the BaCO_3_ powders by AMS. Liquid H_3_PO_4_ (5 mL) was poured into the left round-bottom portion of the gas-tight glass tube, and BaCO_3_ (20 mg) was put into the right round-bottom section. This tube was connected to the vacuum manifold line under reduced pressure (<10^−2^ mbar) for 24 hours at room temperature to remove any remaining water in the H_3_PO_4_ liquid. The H_3_PO_4_ liquid was slowly transferred to the right bottom including the BaCO_3_ powders. Additional liquid was transferred if the reaction was not active. After all the liquid was moved, this tube was incubated in a hot water bath (ca. 90 °C) for 1 hour. Subsequently, the CO_2_ and H_2_O were cold-trapped into another tube using dry ice-ethanol (−76 °C) connected to the closed vacuum line system. The cold-trap step was repeated twice. Only pure CO_2_ was cold-trapped in a quartz tube with pure ferrous powder, using liquid nitrogen (−196 °C) from the reactants such as CO_2_, and H_2_O in another tube under dry ice-ethanol. This CO_2_ with hydrogen and the ferrous powder was reduced to graphite at 650 °C for 10 hours. After these processes, pure graphite with oxidized iron (1 mg) was transferred to a sample holder (small rod shape; 1 mm hole) as indicated in Figure 4 (right-bottom picture).

### 2.5. Measurement of Percent Modern Carbon

**Figure 5.**
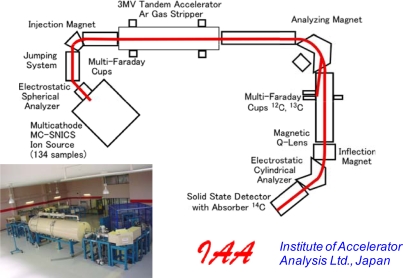
Outline of AMS apparatus (size ca. 15 × 10 m, height 2 m) for determining the percent modern carbon (pMC) by the ratio of ^14^C/^12^C (^14^As) at Institute of Accelerator Analysis Ltd., Japan.

The measurement of the ratio of the three carbon isotopes (^14^C, ^13^C and ^12^C) using Accelerator Mass Spectroscopy (AMS) was performed at IAA as outlined in Figure 5. The carbon in graphite, transferred from the BaCO_3_ samples, was ionized using a cesium cation beam. The anionized carbons were accelerated using a 3MV tandem accelerator (NEC Pelletron, 9SDH-2). The accelerated carbon isotopes were separated by an analyzing magnet based on the different atomic masses. The amounts of ^12^C and ^13^C were detected as a current using multi-Faraday cups. The ^14^C atoms were detected using a solid state detector with a semiconductor absorber. The ratio of ^14^C to ^12^C for the tested sample (^14^As) obtained from the analysis of the BaCO_3_ powders was calculated from the measured amounts of ^14^C and ^12^C. The percent of modern carbon (pMC) for an oil-based carbon is 0%. The pMC for a biomass made by the fixation of CO_2_ in the modern atmosphere through photosynthesis is 108–110%. A measurement of a product’s ^14^As(^14^C/^12^C) content is determined relative to the ratio of ^14^C to ^12^C for reference material (^14^Ar) such as the modern carbon-based oxalic acid radiocarbon (Standard Reference Material (SRM) 4990c, the National Institute of Standards and Technology, NIST, USA).

The pMC values for determining the CO_2_ (respiration) were estimated by tertiary curve fitting for the measured pMC values as shown in Table 2.

## 3. Results and Discussion

### 3.1. Biodegradation of PBS Evaluated from the Blank and Sample Vessels Based on ISO 14855-2

The method used for the determination of the biodegradability of the PBS powders was based on the International Standard (ISO 14855-2) that measures the evolved CO_2_ amount from both the blank vessel without a sample and the sample vessel including a 10 g PBS powder sample, 144 g mature compost, and 320 g sea sand.

The newly developed biodegradation measurement system using the MODA apparatus with the absorption columns is shown in Figure 2. This evaluation system for the biodegradation uses the CO_2_ trap system with CO_2_ absorption columns. This MODA mechanism is as follows. First, room air is passed into the carbon dioxide trap to remove the CO_2_ in the air as shown in Figure 2. This air is moisturized and passed into the reaction vessel controlled at 58 °C using a thermosensor and ribbon heater. The air with the produced CO_2_ from the biodegradation of the samples and respiration of the microorganisms in the compost is passed into the ammonia trap to remove the produced ammonia from the compost to obtain an accurate carbon balance using a gravimetric measurement. The air with its CO_2_ is passed into dehumidifying traps to remove the moisture from the air stream for an accurate carbon weight balance and then passed into an absorption column for the carbon dioxide and an absorption column for water. In these two columns with soda lime (NaOH immobilized in flaked lime) and soda talc (NaOH immobilized in talc), the produced CO_2_ is completely absorbed by the reactions indicated by Equation (2):

(2) $${\text{CO}}_{2}+2\text{NaOH}\to {\text{Na}}_{2}{\text{CO}}_{3}+{\text{H}}_{2}\text{O}$$

The H_2_O produced by this reaction is simultaneously trapped in these two columns. According to this reaction, the weight of these two columns is increased by the same amount as the weight of the produced CO_2_. In this way, the produced CO_2_ amount is easily obtained by a gravimetric measurement. Once a day, the weights of these two columns are measured. From the increasing weight of these two columns for the sample and blank, and the theoretical CO_2_ amount, the percent of biodegradation can be calculated.

**Figure 6.**
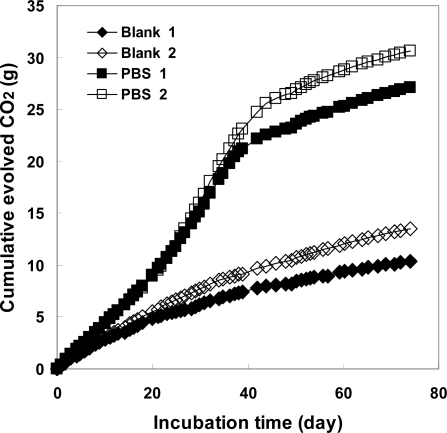
Evolved CO_2_ from sample reaction vessel (500 mL) (including 10 g PBS, 60 g compost and 360 g sea sand) and blank vessel (500 mL) (60 g compost and 360 g sea sand) at 58 °C for PBS biodegradation under controlled compost based on ISO 14855-2.

The PBS powders were almost degraded in the controlled compost at 58 °C using the MODA apparatus. The cumulative evolved CO_2_ amounts of the blank and sample vessels were 11.96 and 28.88 g after a 74-day incubation time, respectively. Figure 6 shows the evolved CO_2_ amounts from the blank and sample vessels. From the beginning of the test period, the difference in the evolved CO_2_ amounts from the blank and sample vessels was very small indicating that PBS was not actively biodegraded during the initial period. During the first 20 days, the difference between the CO_2_ from the blank and that from sample was 3.8 g, indicating that 19% of the PBS was biodegraded. The theoretical evolved CO_2_ from 10 g of PBS is 20.47 g. The determined biodegradability of PBS at 74 days was 82.27% [= (28.88 – 11.96)/20.47 × 100]. Figure 8 shows the calculated biodegradability of PBS determined by the ISO and AMS methods. In this way, the PBS powder was gradually biodegraded in the controlled compost at 58 °C according to the ISO method. PBS is a biodegradable polyesters, that dose not exist in the natural environment and is an artificial copolyester with the ABAB structure (an alternating copolyester). Active degradation enzymes for PBS are available as commercial isolated enzyme [21], filamentous fungus [24] or yeast [25]. However, under general composting conditions, there is no special biodegradation enzyme for PBS as in the soil environment [26]. In this way, PBS is gradually biodegraded by general lipases and changes to CO_2_ or metabolites via an oligomer or monomer such as 1,4-butanediol and succinic acid of PBS.

### 3.2. Biodegradability Determined in One Compost Vessel without a Blank Vessel Using Percent Modern Carbon Determined by AMS

For the biodegradability determination of a sample based on the International Standard, the evolved CO_2_ amount from a blank vessel including the compost inoculum is necessary, as already described, because the amount of evolved CO_2_ from the sample vessel includes the respired CO_2_ and CO_2_ biodegraded from the PBS sample. To calculate only the CO_2_ amount from the PBS biodegradation, the evolved CO_2_ amount from the blank is subtracted from that of the sample vessel. Therefore, the respiration activity in the reaction vessel in the presence of the sample is speculated to be the same as that of the blank reaction vessel without the sample.

There is a method of determining the ratio of CO_2_ from the compost which is biomass and that biodegraded from PBS which is produced from petroleum during the evaluation period of the biodegradation. Biomass carbon, such as the compost produced from agricultural waste, includes ^14^C radio carbon atoms which have been photosynthesized from CO_2_ in the modern atmosphere. The percent modern carbon (pMC) is relative to the ^14^C concentration based on oxalic acid as a standard reference material (SRM 4990c) measured by AMS as indicated by Equations (3) and (4). From the pMC value of evolved CO_2_, the ratio of the evolved CO_2_ from the compost including ^14^C, which is at the biomass level (ca. 1 × 10^−12^), and that from PBS, which is at the petroleum level (no ^14^C), can be determined. The pMC of PBS used in this paper was 0%.

(3) $$\Delta^{14}\text{C}=[{(}^{14}\text{As}{-}^{14}\text{Ar}{/}^{14}\text{Ar}]\times 1000\;\;(‰)$$

(4) $$\text{pMC}=\Delta^{14}\text{C}/10+100\;\;\;\;\;\;\;\;\;\;\;\;\;\;\;\;\;\;\;\;\;\;\;\;\;\;\;\;(\%)$$

where ^14^As and ^14^Ar are the ratio of the ^14^C to ^12^C for the sample and reference (SRM 4990c), respectively. Table 2 indicates the evolved CO_2_ amounts from one vessel, and the pMC of the trapped CO_2_ in BaCO_3_ measured by AMS. In the AMS method, the total evolved CO_2_ amount is the sum of the CO_2_ amount in BaCO_3_ and the trapped CO_2_ in the absorption columns after the Ba(OH)_2_ trap bottle as indicated in Figure 2. The CO_2_ (respiration) and CO_2_ (biodegradation) values were calculated as follows:

(5) $${\text{CO}}_{2}\;(\text{respiration})\;\text{value}\;=\;\text{total}\;\text{evolved}\;{\text{CO}}_{2}\times \frac{\text{pMC}\;(\text{sample})}{\text{pMC}\;(\text{compost})}$$

(6) $${\text{CO}}_{2}\;(\text{biodegradation})\;\text{value}\;=\;\text{total}\;\text{evolved}\;{\text{CO}}_{2}\;\times \frac{\text{pMC}\;(\text{compost})-\text{pMC}\;(\text{sample})}{\text{pMC}\;(\text{compost})}$$

The pMC (compost) value was 109.87 measured by AMS for the trapped BaCO_3_ from different blank vessels for 0–3 days. Indeed, this value is constant when using the same compost, and that of the ordinary compost is around 108, which is the biomass value. In these AMS methods, the measurement of pMC (compost) is not necessary if the same compost is used as the inoculum. These respiration and biodegradation CO_2_ values can be calculated from only one reaction vessel. The evolved CO_2_ value from the sample for the ISO method and the total CO_2_ value for the AMS method were measured under the same compost conditions using a CO_2_ trap apparatus which included the absorption columns and a Ba(OH)_2_ bottle. At the end of the test period (74 days), the total evolved CO_2_ amount in the ISO method was 28.88 g, and the total CO_2_ amount in the AMS method was 28.82 g. Based on these results, the evolved CO_2_ was completely trapped without any loss in the AMS method.

In this experiment during the PBS biodegradation, the measured pMC of the trapped BaCO_3_ gradually decreased to 42.98% after a 49-day incubation time from 109.87% [pMC (compost)]. The measured pMC value of the trapped BaCO_3_ during 60–74 days had still not recovered to the value of the compost, when the biodegradation was almost finished based on the ISO method. In addition, the total CO_2_ (respiration) amount was 17.32 g. This value was higher than that (11.96 g) of the blank vessels based on the ISO method as indicated in Figure 6. For the PCL biodegradation in the controlled compost at 58 °C, 80% of the PCL was well-degraded after 20 days [11]. The pMC values of the evolved CO_2_ from the sample vessel with the PCL ranged from 20 to 30 in this period. However, for the PBS in this experiment, a 37% of the evolved CO_2_ was still the respiration during the 38–60 days. This may be due to not only respiration, but also metabolism of the microorganisms in the compost.

**Figure 7.**
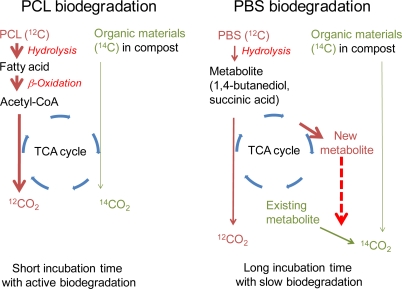
Metabolic pathways for polycaprolactone (PCL) and PBS biodegradation by microorganisms living in controlled compost. ^12^C: Carbon with no ^14^C from petroleum-based material such as PCL and PBS; ^14^C: Carbon with natural occurring ^14^C from biomass, such as the compost.

PBS is biodegraded slower than PCL. In addition, the intermediate materials during the PBS biodegradation were 1,4-butanediol and succinic acid monomer which are components or related metabolites in the TCA cycle. Figure 7 shows the metabolic pathways of the PCL and PBS biodegradations by microorganisms living in the controlled compost. For the PBS biodegradation, changing from the already present metabolite to the newly produced metabolite from PBS occurs in microorganisms in compost. The old metabolites are then metabolized to CO_2_ which is evolved with ^14^C at the natural level during the PBS biodegradation without ^14^C. Part of the carbon from PBS is changed to CO_2_, while others are changed to biomass (metabolites), when PBS was biodegraded to organic compounds in the compost. For the PCL, the biodegraded monomer unit is hydroxycaproic acid. This material may be easily incorporated into the β-oxidation cycle. Therefore, acetyl-CoA may be actively produced. CO_2_ is actively evolved from PCL via the TCA cycle from acetyl-CoA.

**Figure 8.**
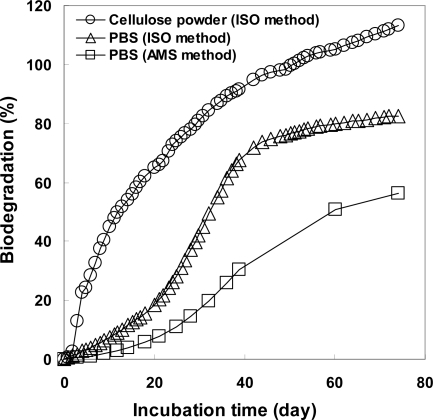
Biodegradabilities of cellulose powder and PBS in controlled compost at 58 °C by ISO method (○, ▵) and AMS method (□).

Figure 8 shows the biodegradabilities of PBS based for the ISO and AMS methods. For the ISO method, the biodegradabilities were calculated by Equation (1) using the data in Figure 6. For the AMS method, the biodegradabilities were calculated from the CO_2_ (biodegradation) and theoretical evolved CO_2_ from 10 g of PBS in each test period as shown in Table 2. It was found that the biodegradabilities determined by the CO_2_ amounts from PBS in the sample vessel were 30% lower than those based on the total CO_2_ amounts from the sample and blank vessels. These differences between the ISO and AMS methods are caused by the carbons from PBS that are changed to metabolites by the microorganisms in the compost, and still not changed to CO_2_. Instead, the already present metabolites inside the microorganisms are changed to CO_2_. This CO_2_ amounts are not counted by the AMS method.

## 4. Conclusions

The biodegradation evaluation methods in a controlled compost at 58 °C based on ISO 14855-2 could be used for the polymers with a slow biodegradation rate such as PBS. For these kinds of slow biodegradable polymers, the powder shape, which has a greater surface area, should be used to confirm its complete biodegradation. For this evaluation, the ISO 14855-2 method is suitable. The exact evaluation of the biodegradation for plastic materials is very important for certification of their environmental aspects.

It was found for the AMS method used in this study, not all the carbon from the slow biodegradable polymer, such as PBS, was changed to produce CO_2_ during the biodegradation in the controlled compost. To clarify the carbon balance of the PBS biodegradation in the compost, it is necessary to separate the remaining PBS, newly produced and existing metabolites in the compost. This is very difficult to be realized to obtain reliable results.

The high cost and highly sophisticated instrumentation prevents a side application of AMS method for the evaluation of the polymers biodegradation. On the contrary, the ISO evaluation methods for the biodegradation have been accepted by many companies and organizations due to the simple methodology. International Standards require clear and simple regulations for such evaluation. It is acceptable for the International Standards that the biodegradabilities of test samples include the effect of the increased respiration and the changing to metabolites in the microorganisms (biomass). In addition, for a wider AMS method application a new analytical protocol needs to be developed for biobased plastics using few ^14^C labeled compounds, this will be the subject of near future investigations of the authors, based on the AMS technique. The biodegradation data obtained from the AMS will be compared to the data from the ISO methods. Therefore, the carbon balance of biodegradation for bioplastics will be clarified. In addition, it will be important to use a scintillation counter for determining the respiration activities by the ^14^C concentration, since this apparatus is wider used than the AMS.

## Figures and Tables

**Table 1.** Properties of the controlled compost.

**Analysis**	**YK-6**
Total dry solids (%)a)	42
Volatile solids (%)b)	56
pH of compost solution	6.9
Total organic carbon amount (%)	9.1
Total nitrogen amount (%)	1.7
C/N ratio	5.0

a) The amount of solids obtained by taking a known volume of compost and drying at about 105 °C for 10 hours;

b) The amount of solids obtained by subtracting the residue of a known volume of compost after incineration at about 550 °C.

**Table 2.** Evolved CO_2_ amounts from sample vessel, percent modern carbon (pMC) and biodegradabilities based on accelerator mass spectrometry (AMS) for poly(butylene succinate) (PBS) biodegradation under controlled compost at 58 °C.

**Test period (day) (center date (day))**	**Evolved CO_2_a) (g) from sample vessel**	**Measured pMCb) (%)**	**Estimated pMCc) (%)**	**CO_2_ amount (g)**		**Biodegradabilityf) (%)**	
				**respirationd)**	**PBS biodegradatione)**	**in period**	**Total**
Blank(0–3)		109.87					
0–3 (1.5)	1.39	91.87	101.95	1.29	0.10	0.5	0.5
3–6 (4.5)	1.14		98.45	1.03	0.11	0.5	1.0
6–9 (8.5)	1.44	97.04	94.81	1.24	0.20	1.0	2.0
9–12 (10.5)	1.12		92.02	0.94	0.18	0.9	2.9
12–14 (13)	0.99		88.19	0.79	0.20	1.0	3.8
14–18 (16)	1.67	83.44	83.20	1.26	0.41	2.0	5.8
18–21 (19.5)	1.42	84.00	77.00	1.00	0.42	2.1	7.9
21–24 (22.5)	1.76	69.05	71.54	1.15	0.61	3.0	10.9
24–28 (26)	1.83		65.21	1.09	0.74	3.6	14.5
28–32 (30)	2.32	51.77	58.29	1.23	1.09	5.3	19.8
32–36 (34)	2.32		52.05	1.10	1.22	6.0	25.8
36–38 (37)	1.67	48.12	48.03	0.73	0.94	4.6	30.4
38–60 (49)	6.67	42.98	40.60	2.46	4.21	20.5	50.9
60–74 (67)	3.08	70.69, 69.53	71.53	2.01	1.07	5.3	56.2
Total	28.82			17.32	11.50		

a) Evolved CO_2_ amounts were measured by CO_2_ weight of BaCO_3_ in additional Ba(OH)_2_ trap and gravimetric method with CO_2_ absorption columns from sample vessel including PBS, compost and sea sand.

b) Measured pMC of graphite transferred from trapped BaCO_3_ by AMS.

c) Estimated pMC was calculated by tertiary curve fitting for measured pMC values. pMC = 0.0009x^3^ – 0.0598x^2^ – 0.5007x + 102.83

x: center date of period (day).

d) CO_2_ (respiration) was calculated according to Equation (5).

e) CO_2_ (biodegradation) was evolved CO_2_ minus CO_2_ (respiration).

f) Biodegradability was CO_2_ (biodegradation) divided by theoretical evolved CO_2_ of 10 g PBS (20.47 g).

## References

[1] Michel V Aliphatic polyesters: Great degradable polymers that cannot do everything Biomacromolecules 2005 6 538 546 15762610 10.1021/bm0494702

[2] Japan BioPlastic Association HomePage. http://www.jbpaweb.net/english/english.htm (accessed September 29, 2009).

[3] Determination of the Ultimate Aerobic Biodegradability of Plastic Materials under Controlled Composting Conditions—Method by Analysis of Evolved Carbon Dioxide—Part 2: Gravimetric Measurement of Carbon Dioxide Evolved In a Laboratory-Scale TestISO 14855-2; ISO Geneva, Switzerland 2007

[4] Karlsson S Ljungquist O Albertsson A-C Biodegradation of polyethylene and the influence of surfactants Polym. Degrad. Stab 1988 21 237 250

[5] Standard Test Methods for Determining Aerobic Biodegradation of Radiolabeled Plastic Materials in an Aqueous or Compost EnvironmentASTM D6340–98ASTM West Conshohocken, PA, USA 1998

[6] Standard Test Method for Determining the Biodegradability of Radiolabeled Polymeric Plastic Materials in Seawater; ASTM D6692–01ASTM West Conshohocken, PA, USA 2001

[7] Standard Test Method for Determining Anaerobic Biodegradability of Radiolabeled Plastic Materials in a Laboratory-Scale Simulated Landfill Environment; ASTM D6776–02ASTM West Conshohocken, PA, USA 2002

[8] Federie TW Bariaz MA Pettigrew CA Kerr KM Kemper JJ Nuck BA Schechtman LA Anaerobic biodegradation of aliphatic polyesters: Poly(3-hydroxybutyrate-co-3-hydroxyoctanoate) and poly(e-caprolactone) Biomacromolecules 2002 3 813 822 12099827 10.1021/bm025520w

[9] Ponsart S Coudane J Saulnier B Morgat J-L Vert M Biodegradation of [^3^H] poly(ɛ-caprolactone) in the presence of active sludge extracts Biomacromolecules 2001 2 373 377 11749195 10.1021/bm015549k

[10] Vert M Santos ID Ponsart S Alauzet N Morgat J-L Coudane J Degradable polymers in a living environment: Where do you end up? Polym. Int 2002 51 840 844

[11] Kunioka M Ninomiya F Funabashi M Novel evaluation method of biodegradabilities for oil-based polycaprolactone by naturally occurring radiocarbon-14 concentration using accelerator mass spectrometry based on ISO 14855-2 in controlled compost Polym. Degrad. Stab 2007 92 1279 1288

[12] Standard Test Methods for Determining the Biobased Content of Solid, Liquid, and Gaseous Samples using Radiocarbon Analysis; ASTM D6866–08ASTM West Conshohocken, PA, USA 2008

[13] Narayan R Biobased and Biodegradable Polymer Materials ACS Polymer Preprints San Diego, CA, USA 2005 319 320

[14] Currie LA Klinedinst DB Burch R Feltham N Dorsch R Authentication and dating of biomass components of industrial materials; links to sustainable technology Nucl. Instrum. Methods Phys.Res. Sect. B 2000 172 281 287

[15] Kunioka M Inuzuka Y Ninomiya F Funabashi M Biobased contents of biodegradable poly(ɛ-caprolactone) composites polymerized and directly molded using aluminium triflate from caprolactone with cellulose and inorganic filler Macromol. Biosci 2006 6 517 523 16832812 10.1002/mabi.200600037

[16] Kunioka M Ninomiya F Funabashi M Biobased contents of organic fillers and polycaprolactone composites with cellulose fillers measured by accelerator mass spectrometry based on ASTM D6866 J Polym Environ 2007 15–4 281 287

[17] Funabashi M Ninomiya F Ohara K Kunioka M Biomass carbon ratio of biomass chemicals measured by accelerator mass spectrometry Bull Chem Soc Jpn(submitted for publication).

[18] Fujimaki T Precessability and properties of aliphatic polyesters, ‘BIONOLLE’, synthesized by polycondensation reaction Polym. Degrad. Stab 1998 59 209 214

[19] Mizukoshi T Bionolle—Approach to the more environmen-tally-friendly green plastics The 2nd International Conference of Technology and Application of Biodegradable and Biobased Plastics (ICTABP2) Hangzhou, China 2006 123

[20] Kato S Tsukuhara T Kishimoto M Nagaya I Development of green sustainable plastic (GS Pla) The 2nd International Conference of Technology and Application of Biodegradable and Biobased Plastics (ICTABP2) Hangzhou, China 2006 96

[21] Shitani N Kato S Green sustainable plastic (GS Pla) and GS Pla-degrading enzyme from fungus The First Asian-Oceanian Conference on Green & Sustainable Chemistry (GSC-AON 2007) Tokyo, Japan 2007 235

[22] Kunioka M Ninomiya F Funabashi M Biodegradation of poly(lactic acid) powders proposed as the reference test materials for the international standard of biodegradation evaluation methods Polym. Degrad. Stab 2006 91 1919 1928

[23] Funabashi M Ninomiya F Kunioka M Biodegradation of polycaprolactone powders proposed as reference test materials for international standard of biodegradation evaluation method J. Polym. Environ 2007 15 7 17

[24] Maeda H Yamagata Y Abe K Hasegawa F Macheda M Ishioka R Gomi K Nakajima T Purification and characterization of a biodegradable plastic-degrading enzyme from Aspergillus oryzae Appl. Microbiol. Biotechnol 2005 67 778 788 15968570 10.1007/s00253-004-1853-6

[25] Masaki K Kamini NR Ikeda H Iefuji H Cutinase-like enzyme from the yeast Cryptococcus sp. strain S-2 hydrolyzes polylactic acid and other biodegradable plastics Appl. Environ. Microbiol 2005 71 7548 7550 16269800 10.1128/AEM.71.11.7548-7550.2005 PMC1287645

[26] Tominaga Y Matsukawa K Kitagawa K Nakayama A Field test of soil biodegradability of biodegradable plastics in Japan The 10th Pacific Basin Conference on Hazardous Waste at Okayama Okayama, Japan 2001 253 256

